# Plasma phosphorylated tau181 outperforms [ ^18^F] fluorodeoxyglucose positron emission tomography in the identification of early Alzheimer disease

**DOI:** 10.1111/ene.16255

**Published:** 2024-10-24

**Authors:** Kely Monica Quispialaya, Joseph Therriault, Antonio Aliaga, Cécile Tissot, Stijn Servaes, Nesrine Rahmouni, Thomas K. Karikari, Andrea L. Benedet, Nicholas J. Ashton, Arthur C. Macedo, Firoza Z. Lussier, Jenna Stevenson, Yi‐Ting Wang, Jaime Fernandez Arias, Ali Hosseini, Takashi Matsudaira, Bertrand Jean‐Claude, Brian M. Gilfix, Eduardo R. Zimmer, Jean‐Paul Soucy, Tharick A. Pascoal, Serge Gauthier, Henrik Zetterberg, Kaj Blennow, Pedro Rosa‐Neto, for the Alzheimer's Disease Neuroimaging Initiative

**Affiliations:** ^1^ Translational Neuroimaging Laboratory McGill University Research Centre for Studies in Aging Douglas Hospital McGill University Montreal Quebec Canada; ^2^ Montreal Neurological Institute Montreal Quebec Canada; ^3^ Department of Experimental Medicine McGill University Montreal Quebec Canada; ^4^ Department of Neurology and Neurosurgery McGill University Montreal Quebec Canada; ^5^ Department of Pharmacology, Graduate Program in Biological Sciences: Biochemistry (PPGBioq) and Pharmacology and Therapeutics (PPGFT) Universidade Federal do Rio Grande do Sul Porto Alegre Brazil; ^6^ Department of Psychiatry and Neurochemistry Institute of Neuroscience and Physiology Sahlgrenska Academy University of Gothenburg Mölndal Sweden; ^7^ Department of Neurology and Psychiatry University of Pittsburgh School of Medicine Pittsburgh Pennsylvania USA; ^8^ Wallenberg Centre for Molecular Medicine University of Gothenburg Gothenburg Sweden; ^9^ Department of Biofunctional Imaging Hamamatsu University School of Medicine Hamamatsu Japan; ^10^ Department of Neurology National Hospital Organization Shizuoka Institute of Epilepsy and Neurological Disorders Urushiyama Japan; ^11^ Department of Specialized Medicine McGill University Montreal Quebec Canada; ^12^ Department of Pharmacology Universidade Federal do Rio Grande do Sul Porto Alegre Brazil; ^13^ Brain Institute of Rio Grande do Sul Pontifícia Universidade Católica do Rio Grande do Sul Porto Alegre Brazil; ^14^ Department of Psychiatry McGill University Montreal Quebec Canada; ^15^ Clinical Neurochemistry Laboratory Sahlgrenska University Hospital Mölndal Sweden; ^16^ Department of Neurodegenerative Disease University College London Institute of Neurology London UK; ^17^ UK Dementia Research Institute at University College London London UK; ^18^ Hong Kong Centre for Neurodegenerative Diseases China; ^19^ Wisconsin Alzheimer's Disease Research Center University of Wisconsin School of Medicine and Public Health University of Wisconsin–Madison Madison Wisconsin USA

**Keywords:** [^18^F]florbetapir‐PET, [^18^F]FDG‐PET, Alzheimer disease, cerebrospinal fluid, plasma p‐tau181

## Abstract

**Background and purpose:**

This study was undertaken to compare the performance of plasma p‐tau181 with that of [^18^F]fluorodeoxyglucose (FDG) positron emission tomography (PET) in the identification of early biological Alzheimer disease (AD).

**Methods:**

We included 533 cognitively impaired participants from the Alzheimer's Disease Neuroimaging Initiative. Participants underwent PET scans, biofluid collection, and cognitive tests. Receiver operating characteristic analyses were used to determine the diagnostic accuracy of plasma p‐tau181 and [^18^F]FDG‐PET using clinical diagnosis and core AD biomarkers ([^18^F]florbetapir‐PET and cerebrospinal fluid [CSF] p‐tau181) as reference standards. Differences in the diagnostic accuracy between plasma p‐tau181 and [^18^F]FDG‐PET were determined by bootstrap‐based tests. Correlations of [^18^F]FDG‐PET and plasma p‐tau181 with CSF p‐tau181, amyloid β (Aβ) PET, and cognitive performance were evaluated to compare associations between measurements.

**Results:**

We observed that both plasma p‐tau181 and [^18^F]FDG‐PET identified individuals with positive AD biomarkers in CSF or on Aβ‐PET. In the MCI group, plasma p‐tau181 outperformed [^18^F]FDG‐PET in identifying AD measured by CSF (*p* = 0.0007) and by Aβ‐PET (*p* = 0.001). We also observed that both plasma p‐tau181 and [^18^F]FDG‐PET metabolism were associated with core AD biomarkers. However, [^18^F]FDG‐PET uptake was more closely associated with cognitive outcomes (Montreal Cognitive Assessment, Mini‐Mental State Examination, Clinical Dementia Rating Sum of Boxes, and logical memory delayed recall, *p* < 0.001) than plasma p‐tau181.

**Conclusions:**

Overall, although both plasma p‐tau181 and [^18^F]FDG‐PET were associated with core AD biomarkers, plasma p‐tau181 outperformed [^18^F]FDG‐PET in identifying individuals with early AD pathophysiology. Taken together, our study suggests that plasma p‐tau181 may aid in detecting individuals with underlying early AD.

## INTRODUCTION

Alzheimer disease (AD) is defined by the aggregation of amyloid β (Aβ) plaques and tau neurofibrillary tangles, which distinguish it from other neurodegenerative diseases resulting in dementia [1, 2]. Clinical diagnosis of AD is difficult, with misdiagnoses ranging from 15% to 30% at expert centres [3, 4] and higher rates at primary care centres. Biomarkers are increasingly used in the differential diagnosis of AD [5, 6] as well as inclusion criteria for therapeutic trials targeting key features of AD [7, 8].

Recently, phosphorylated tau (p‐tau) in plasma biomarkers have demonstrated high sensitivity and specificity for AD pathologies, and strong performance in distinguishing AD from other neurodegenerative diseases [9, 10, 11, 12, 13, 14]. Because of their specificity for AD, plasma p‐tau biomarkers could transform the diagnosis of AD by providing affordable and scalable diagnostic tools [15, 16]. The most widely employed diagnostic biomarker for AD is [^18^F]fluorodeoxyglucose (FDG) positron emission tomography (PET), which provides topographical information about synaptic function as indexed by brain glucose metabolism, which is assessed visually in the differential diagnosis of AD [17]. Although [^18^F]FDG‐PET is widely used in the differential diagnosis of cognitive impairment, [^18^F]FDG‐PET patterns are not specific for AD [18], as multiple neurodegenerative diseases can result in a pattern of glucose hypometabolism usually associated with AD.

The goal of this study is, therefore, to assess whether p‐tau in plasma provides superior diagnostic capability to [^18^F]FDG‐PET in the identification of biological and clinical AD. Here, we compare the diagnostic performance of plasma p‐tau181 and [^18^F]FDG‐PET in the Alzheimer's Disease Neuroimaging Initiative (ADNI) cohort and compare the relationships of both biomarkers with clinical symptoms.

## MATERIALS AND METHODS

### Study design

The data were obtained from the ADNI database (https://adni.loni.usc.edu/). ADNI is a multicentre study launched in 2003 as a public–private partnership, led by Principal Investigator Michael W. Weiner, MD. ADNI recruits participants at 57 sites in the USA and Canada. The primary goal of ADNI is to test whether the combination of neuroimaging and biochemical biomarkers and clinical and neuropsychological assessments can be used for early detection and monitoring of AD dementia. The ADNI study was approved by local institutional review boards of participating institutions, and informed written consent was provided by enrolled participants at each site. The data used were downloaded in November 2021. Full information regarding the ADNI inclusion/exclusion criteria is described elsewhere (https://adni.loni.usc.edu/).

### Participants

Study participants were stratified based on clinical diagnosis. We included participants with mild cognitive impairment (MCI; *n* = 410) and patients with probable AD (pAD; *n* = 123). ADNI criteria for MCI were (i) subjective memory complaints reported by themselves, study partner, or clinician; (ii) objective memory loss defined as scoring below an education‐adjusted cutoff score on delayed recall on the Wechsler Memory Scale‐Revised (WMS‐R) Logical Memory Test (score = 8 for those with 16 years of education, score = 4 for those with 8–15 years of education, score = 2 for those with 0–7 years of education); (iii) global Clinical Dementia Rating (CDR) score of 0.5; and (iv) general cognitive and functional performance sufficiently preserved such that a diagnosis of dementia could not be made by the site physician at the time of screening. ADNI criteria for pAD were (i) subjective memory complaints reported by themselves, study partner, or clinician; (ii) objective memory loss defined as scoring below an education‐adjusted cutoff score on delayed recall of the WMS‐R Logical Memory Test (score = 8 for those with 16 years of education, score = 4 for those with 8–15 years of education, score = 2 for those with 0–7 years of education); (iii) global CDR score of 1 and more; and (iv) individuals had mild AD and had to meet the National Institute of Neurological and Communicative Disorders and Stroke–Alzheimer's Disease and Related Disorders Association criteria for probable AD [19, 20].

The characteristics of study participants are detailed in Table 1. Patients were further stratified according to their biomarker profile as carriers of AD pathophysiology based on cerebrospinal fluid (CSF) or PET [^18^F]florbetapir (see criteria described below).

**TABLE 1:** Demographic and clinical characteristics of the sample.

Characteristic	Overall	MCI	pAD	*p*
Number of patients	533	410	123	‐
Male, *n* (%)	297 (56)	225 (55)	72 (59)	‐
Age, years, median (IQR)	72.20 (66.40–77.50)	71.40 (65.98–76.60)	75.10 (68.30–80.10)	0.0004
Education, years, median (IQR)	16.00 (14.00–18.00)	16.00 (14.00–18.00)	16.00 (14.00–18.00)	0.06
MMSE score, median (IQR)	28 (25–29)	29 (27–29)	23 (21–25)	<0.0001
MoCA score, median (IQR)	28 (25–29)	23 (21–25)	19 (14–21)	<0.0001
CDR‐SB score, median (IQR)	1.5 (1.0–3.0)	1.5 (1.0–2.0)	4.5 (3.00–5.50)	<0.0001
LDEL score, median (IQR)	6.00 (2.00–9.00)	8.00 (5.00–9.00)	1.00 (0.00–3.00)	<0.0001
CSF Aβ42, median (IQR)	817.7 (610.4–1274)	914.1 (676.50–1397)	622.50 (474.90–785.80)	<0.0001
CSF p‐tau181, median (IQR)	25.23 (17.89–36.24)	22.78 (16.36–32.42)	33.95 (25.69–46.04)	<0.0001
CSF Aβ42 + p‐tau181 positivity, *n* (%)^a^		145 (35%)	94 (76%)	
Plasma p‐tau181, median (IQR)	17.80 (11.60–24.10)	15.59 (10.64–22.72)	23.04 (17.77–28.34)	<0.0001
[^18^F]FDG‐PET SUVR, median (IQR)	1.23 (1.13–1.32)	1.27 (1.18–1.35)	1.08 (0.97–1.16)	<0.0001
[^18^F]Florbetapir‐PET SUVR, median (IQR)	1.25 (1.04–1.46)	1.17 (1.02–1.39)	1.43 (1.28–1.58)	<0.0001
[^18^F]Florbetapir‐PET positivity, *n* (%)^b^		227 (55.4%)	109 (88.6%)	

*Note*: Demographic and clinical characteristics for participants in the cross‐sectional dataset are presented, stratified by clinical diagnosis. Number and percentage are reported for categorical variables, whereas continuous variables are reported using median and IQR. Statistical differences between the MCI and pAD groups based on clinical diagnosis were tested for the dataset, performing *t*‐test for categorical variables and Mann–Whitney *U*‐test for nonnormal continuous variables. There was a statistically significant difference across the MCI and pAD groups based on clinical diagnosis (*p* < 0.0001), except for education (when comparing MCI and pAD groups).

Abbreviations: Aβ, amyloid β; CDR‐SB, Clinical Dementia Rating Sum of Boxes; CSF, cerebrospinal fluid; FDG, fluorodeoxyglucose; IQR, interquartile range; LDEL, logical memory delayed recall total; MCI, mild cognitive impairment; MMSE, Mini‐Mental State Examination; MoCA, Montreal Cognitive Assessment; pAD, probable Alzheimer disease; PET, positron emission tomography; SUVR, standardized uptake value ratios.

^a^ CSF Aβ42 + p‐tau181 positivity: Aβ42 < 977 pg/mL as well as p‐tau181 > 23 pg/mL.

^b^ Florbetapir‐PET SUVR positivity > 1.11.

Participants underwent measures of plasma p‐tau181, CSF p‐tau181, [^18^F]FDG‐PET, [^18^F]florbetapir‐PET, and cognitive screening tests. All of these biomarkers were taken from ADNI tables. Plasma p‐tau181 and [^18^F]FDG‐PET data scans were matched with CSF p‐tau181 and [^18^F]florbetapir‐PET at the same ADNI study visit; a description of the sample selections can be found in Additional File 1.

### Standard protocol approvals, registration, and patient consents

The ADNI study was approved by the institutional review boards of each participating institution. Informed written consent was obtained from all participants enrolled in this study (https://adni.loni.usc.edu/).

### Plasma p‐tau181 measurement

Blood samples were collected, shipped, and stored as described by the ADNI Biomarker Core Laboratory (http://adni.loni.usc.edu/methods/). Plasma p‐tau181 was analyzed with the single molecule array (Simoa) technique, using a clinically validated in‐house assay described previously [9]. Plasma p‐tau181 was measured on Simoa HD‐X instruments (Quanterix, Billerica, MA, USA) in April 2020 at the Clinical Neurochemistry Laboratory, University of Gothenburg, Mölndal, Sweden. Plasma p‐tau181 data were collected over 47 analytical runs as described previously [21, 22]. The assay precision was assessed by measuring two different quality control samples at the start and end of each run, resulting in within‐run and between‐run coefficients of variation of 3.3%–11.6% and 6.4%–12.7%, respectively. Of 3762 ADNI plasma p‐tau181 samples, four were removed due to inadequate volumes. The remaining 3758 all measured above the assay's lower limit of detection (0.25 pg/mL), with only six below the lower limit of quantification (1.0 pg/mL). Plasma p‐tau181 measurements were downloaded from the ADNI database.

### CSF measurements

CSF samples were collected by lumbar puncture, shipped, and stored as described by the ADNI Biomarker Core Laboratory (http://adni.loni.usc.edu/methods). CSF concentrations of Aβ42 and p‐tau181 were quantified using fully automated Elecsys immunoassays (Roche Diagnostics, Rotkreuz, Switzerland) at the ADNI Biomarker Laboratory at the University of Pennsylvania. The lower and upper technical limits for CSF p‐tau181 were 8 and 120 pg/mL and for Aβ42 were 200 and 1700 ng/L. Procedures have been described in detail previously [23].

### PET acquisition and processing

A detailed description of amyloid‐PET ([^18^F] florbetapir) and [^18^F]FDG‐PET image acquisition has been described previously (http://adni.loni.usc.edu/methods/pet‐analysis‐method/pet‐analysis). The [^18^F]FDG and [^18^F]florbetapir standardized uptake value ratio (SUVR) maps were generated using the pons and the full cerebellum as the reference region, respectively. [^18^F]FDG‐PET measures include average SUVR of angular gyrus, posterior cingulate, and inferior temporal cortices, which are frequently affected in AD^25^ and predict conversion from MCI to AD dementia [24]. To confirm that these regions were the most relevant for identifying amyloid‐positive versus amyloid‐negative groups, we conducted a voxelwise analysis comparing amyloid‐PET‐positive versus amyloid‐PET‐negative individuals, correcting for age, sex, and education (Additional File S2). [^18^F]Florbetapir‐PET measures included average SUVR of precuneus and prefrontal, orbitofrontal, parietal, temporal, anterior, and posterior cingulate cortices [26].

### Cognitive tests

Cognitive performance was assessed using Mini‐Mental State Examination (MMSE), Montreal Cognitive Assessment (MoCA), CDR Sum of Boxes (CDR‐SB), and logical memory delayed recall total number of story units recalled (LDEL_total).

### Biomarker cutoff points

Based on preestablished AD biomarker thresholds, Aβ positivity was determined for each individual by a global [^18^F]florbetapir‐PET SUVR exceeding 1.11 [27], and brain glucose abnormality was defined as having [^18^F]FDG‐PET SUVR < 1.2 [25]. The threshold for CSF p‐tau181 positivity (>23 pg/mL) also has been previously described [28, 29].

### Statistical analyses

All data analyses were performed using Prism 7.0 software (GraphPad Software, San Diego, CA, USA) and R version 3.6.3 software. First, descriptive statistics to compare demographic variables and clinical characteristics across diagnostic groups were calculated by performing the unpaired *t*‐test for normally distributed continuous variables and Mann–Whitney *U*‐test for nonnormal continuous variables. Participant groups were characterized using median and interquartile range (IQR). The dataset was not normally distributed for fluid biomarkers and neuroimaging biomarkers.

Second, to assess the accuracy of plasma p‐tau181 and [^18^F]FDG‐PET in identifying positive AD biomarkers such as CSF p‐tau181 and Aβ‐PET, receiver operating characteristic (ROC) curves were computed across each diagnostic group individually. Furthermore, the specificity was determined when sensitivity was fixed at 85%, both for plasma p‐tau217 and for [^18^F]FDG‐PET.

Statistical assessment of whether plasma p‐tau181 and [^18^F]FDG‐PET diagnostic accuracies were significantly different was determined by a bootstrap‐based test implemented in the pROC package in R, a statistical framework for comparing two correlated (or paired) ROC curves [30].

Third, Spearman partial correlations between [^18^F]FDG‐PET or plasma p‐tau181 and CSF p‐tau181 or Aβ‐PET adjusted by age and sex were evaluated. Furthermore, Spearman partial correlations between[^18^F]FDG‐PET or plasma p‐tau181 and cognitive tests adjusted by age, sex, and education were evaluated. Statistical evaluation of whether correlation coefficients were significantly different was carried out in R using the Cocor package [31], a statistical framework for comparing associations between intercorrelated measurements. The *p*‐value significance level was 0.05, and all tests were two‐sided.

## RESULTS

### Study participant characteristics

The demographic and clinical characteristics of the study participants are summarized in Table 1. A total of 533 individuals were included in the cross‐sectional dataset; 410 were classified into the MCI group and 123 into the pAD group. Of the 533 participants, 297 (56%) were male and median age was 72.4 years (IQR = 67.0–77.4). There was a significant age difference between the MCI and pAD dementia groups (*p* = 0.0004). In terms of years of education, there was no significant difference between the MCI and pAD (*p* = 0.06) groups. As expected, the pAD dementia group had significantly worse performance on the cognitive tests MMSE (*p* < 0.0001), MoCA (*p* < 0.0001), CDR‐SB (*p* < 0.0001), and LDEL_total (*p* < 0.0001) compared to the MCI individuals.

**FIGURE 1:**
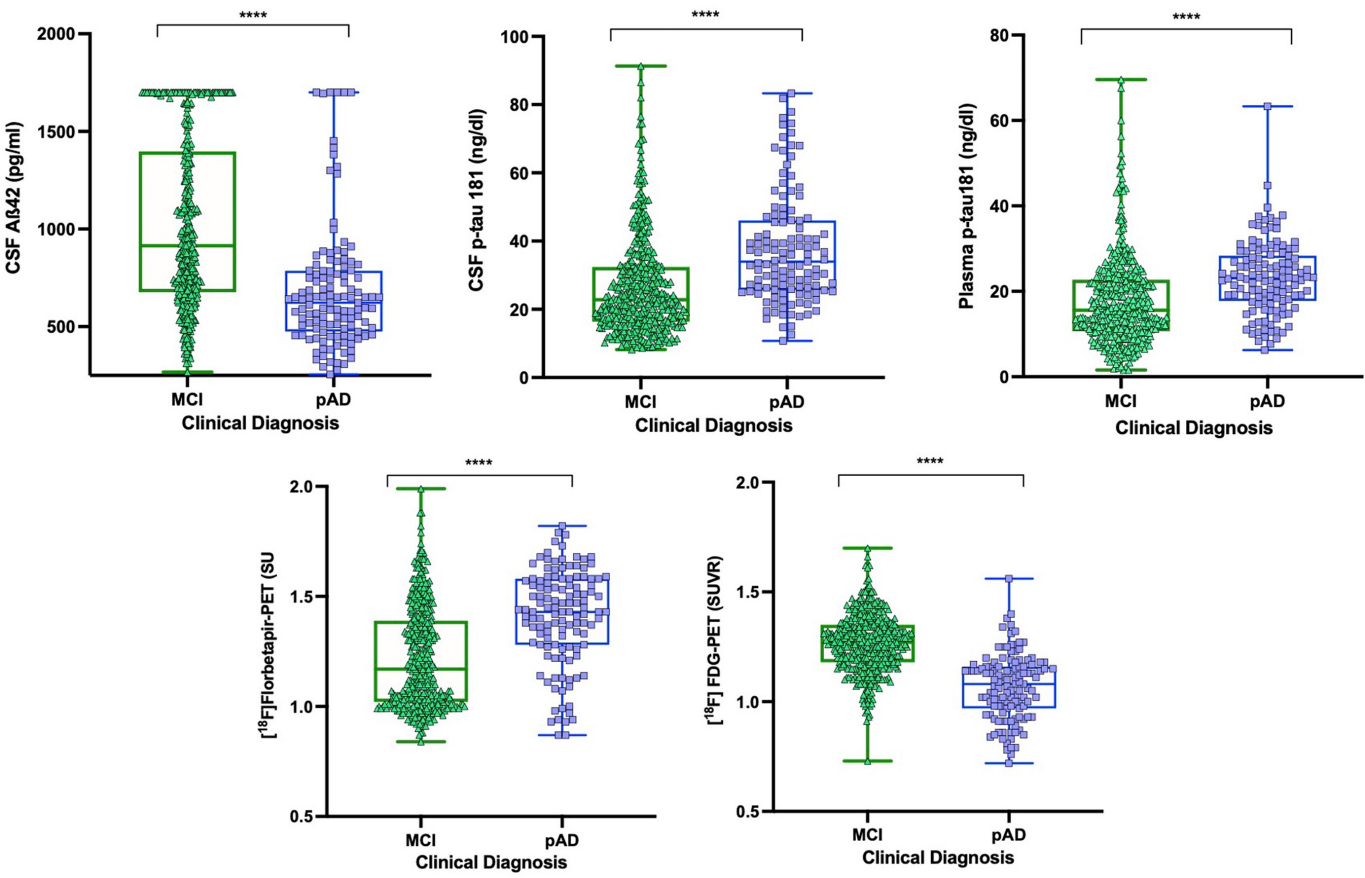
Fluid biomarkers and neuroimaging across clinically defined diagnostics groups. Distribution of cerebrospinal fluid (CSF) p‐tau181 (pg/mL), CSF amyloid β (Aβ) 42 (pg/mL), plasma p‐tau181 (pg/mL), [^18^F] fluorodeoxyglucose (FDG) positron emission tomography (PET) standardized uptake value ratio (SUVR), and [^18^F]florbetapir‐PET SUVR between the clinically defined diagnostics groups mild cognitive impairment (MCI) and probable Alzheimer disease (pAD), using Mann–Whitney *U*‐test, is shown. Statistically significant differences in fluid biomarkers (CSF p‐tau181, CSF Aβ42, and plasma p‐tau181) were found between the MCI and pAD groups (****p < 0.0001). Similarly, significant differences in neuroimaging ([^18^F]FDG‐PET SUVR and [^18^F]florbetapir‐PET SUVR) were found between the MCI and pAD groups (****p < 0.0001).

In regard to fluid biomarkers, individuals with pAD had lower concentrations of CSF Aβ42 (*p* < 0.0001) and significantly elevated (*p* < 0.0001) levels of CSF p‐tau181 compared with the MCI individuals (Table 1, Figure 1). Plasma p‐tau181 concentrations were also highly elevated in pAD individuals compared with the MCI group (*p* < 0.0001; Table 1, Figure 1). The distribution of plasma p‐tau181 according to the clinical group and amyloid‐PET status is reported in Additional File 3. Based on neuroimaging biomarkers status, the pAD group showed higher [^18^F]florbetapir‐PET SUVR (*p* < 0.0001) and lower brain metabolism indexed by [^18^F]FDG‐PET SUVR (*p* < 0.0001) across the diagnostics groups (Table 1, Figure 1).

### Diagnostic performance of plasma p‐tau181 and [^18^ F]FDG SUVR biomarkers

**FIGURE 2:**
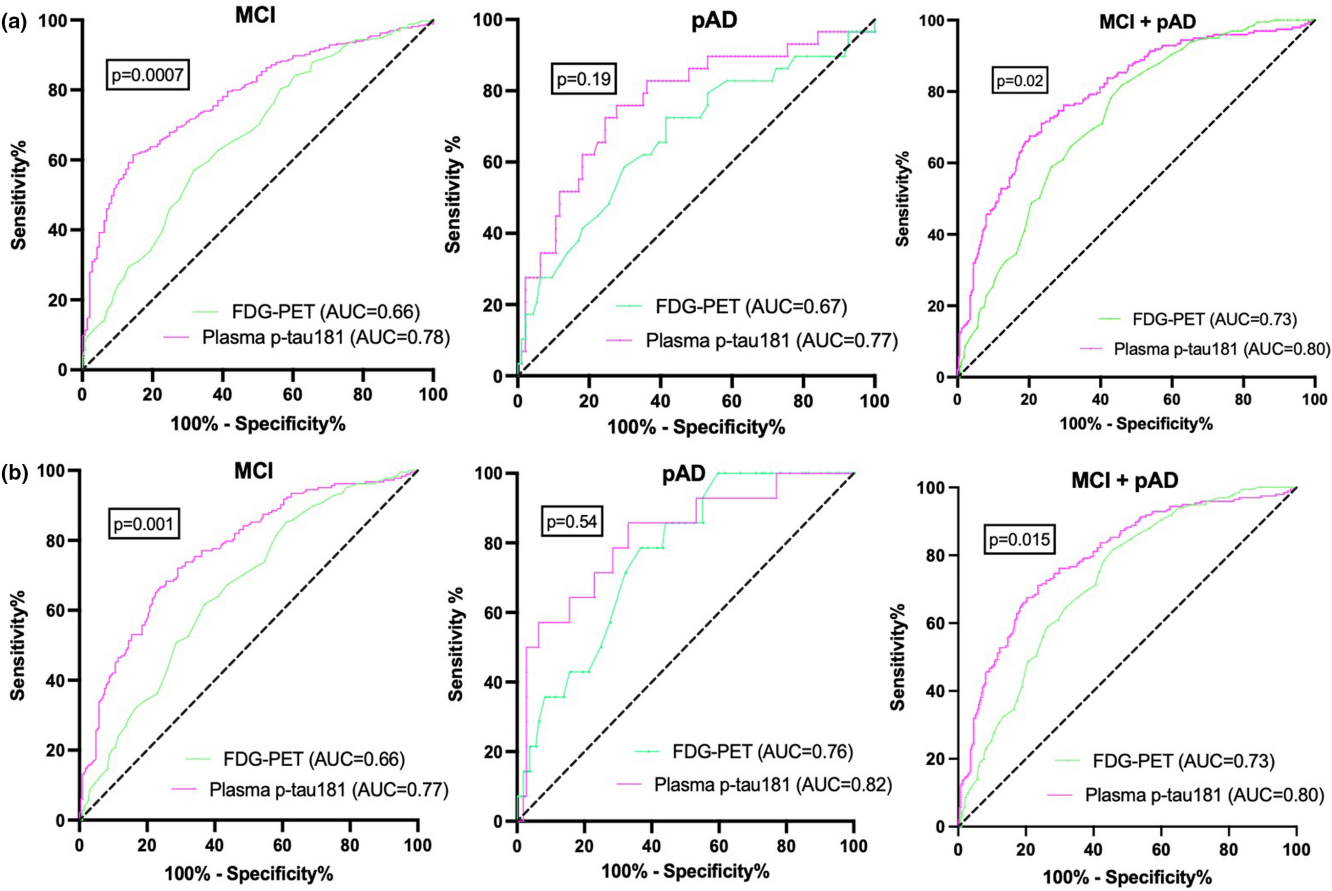
Receiver operating characteristic (ROC) curves for discrimination of patients with cerebrospinal fluid (CSF) and amyloid β positron emission tomography (PET) positivity. ROC curve analysis for the detection of Alzheimer disease pathophysiology is shown. (a) ROC curve analyses for detection of CSF positivity according to established thresholds across each diagnostic group individually and all clinical groups. (b) ROC curve analyses for detection of amyloid‐PET positivity according to established threshold across the different diagnostic groups. The *p*‐value for the difference between the three ROC curves was obtained using a bootstrap‐based method implemented in the pROC package in R (http://www.r‐project.org). AUC, area under the curve; MCI, mild cognitive impairment; pAD, probable Alzheimer disease; FDG, [^18^F] fluorodeoxyglucose.

According to established CSF biomarker thresholds, 239 (45%) individuals had CSF biomarker evidence of biological AD (Aβ1–42 < 977 pg/mL as well as p‐tau181 > 23 pg/mL; Table 1). As for imaging, 336 (63%) individuals were amyloid‐PET‐positive across the entire sample (Table 1). ROC curves were employed to assess the accuracy of plasma p‐tau181 and [^18^F]FDG‐PET in identifying positive AD biomarkers (CSF and Aβ‐PET) across each diagnostic group (Figure 2). In the MCI group, plasma p‐tau181 showed good performance in differentiating participants with positive CSF test (Aβ1–42 < 977 pg/mL and p‐tau181 > 23 pg/mL) from individuals with negative CSF with an area under the curve (AUC) of 0.78 (95% confidence interval [CI] = 0.74–0.83), sensitivity = 86%, specificity = 54%, compared with [^18^F]FDG SUVR with an AUC of 0.66 (95% CI = 0.61–0.72, *p* = 0.0007) sensitivity = 86%, specificity = 47% (Figure 2a). Furthermore, in the pAD group, plasma p‐tau181 differentiated individuals with positive CSF tests from individuals with negative CSF tests with an AUC of 0.77 (95% CI = 0.67–0.87), sensitivity = 86%, specificity = 52%, and [^18^F]FDG SUVR distinguished individuals with positive CSF tests from individuals with negative CSF tests with an AUC of 0.67 (95% CI = 0.55–0.77), sensitivity = 83%, specificity = 39% (Figure 2a). No statistical difference (*p* = 0.19) between plasma p‐tau181 and [^18^F]FDG‐PET was observed for classifying distinguishing individuals with CSF+ tests from individuals with CSF− tests in the pAD group. Across the entire sample (MCI + pAD), plasma p‐tau181 accurately distinguished individuals with CSF+ tests from individuals with CSF− tests with an AUC of 0.80 (95% CI = 0.76–0.83), sensitivity = 85%, specificity = 54%, and [^18^F]FDG SUVR differentiated subjects with CSF+ tests from individuals with CSF− tests with an AUC of 0.73 (95% CI = 0.68–0.76), sensitivity = 85%, specificity = 47% (Figure 2a). A bootstrap method test revealed that there was a small difference (*p* = 0.02) between plasma p‐tau181 and [^18^F]FDG‐PET when distinguishing individuals with CSF+ tests from individuals with CSF− tests.

In regard to the MCI group, plasma p‐tau181 distinguished participants with Aβ‐PET+ from individuals with Aβ‐PET− with an AUC of 0.77 (95% CI = 0.72–0.81), sensitivity = 85%, specificity = 47%, and [^18^F]FDG‐PET SUVR distinguished participants with Aβ‐PET+ from individuals with Aβ‐PET− with an AUC of 0.66 (95% CI = 0.61–0.71), sensitivity = 85%, specificity = 39% (Figure 2b). There was a significant difference between plasma p‐tau181 and [^18^F]FDG‐PET for distinguishing individuals with MCI with Aβ‐PET+ versus Aβ‐PET− subjects (*p* = 0.001).

In the pAD group, [^18^F]FDG‐PET SUVR distinguished participants with Aβ‐PET+ from individuals with Aβ‐PET− with an AUC of 0.76 (95% CI = 0.65–0.87), sensitivity = 86%, specificity = 56%. Plasma p‐tau181 distinguished between Aβ‐PET+ and Aβ‐PET− individuals with pAD with an AUC of 0.82 (95% CI = 0.70–0.94), sensitivity = 85%, specificity = 67% (Figure 2b). There was no statistically significant difference between these two biomarkers ([^18^F]FDG‐PET SUVR and plasma p‐tau181) for classifying participants with Aβ‐PET+ from individuals with Aβ‐PET− with pAD (*p* = 0.54). Plasma p‐tau181 had superior performance in identifying individuals with Aβ‐PET+ from individuals with Aβ‐PET− with an AUC of 0.80 (95% CI = 0.76–0.86), sensitivity = 85%, specificity = 55%, compared with [^18^F]FDG SUVR with an AUC of 0.73 (95% CI = 0.68–0.77), sensitivity = 86%, specificity = 47% (Figure 2b) across the entire sample (MCI + pAD, *p* = 0.001).

### Correlation analysis

**FIGURE 3:**
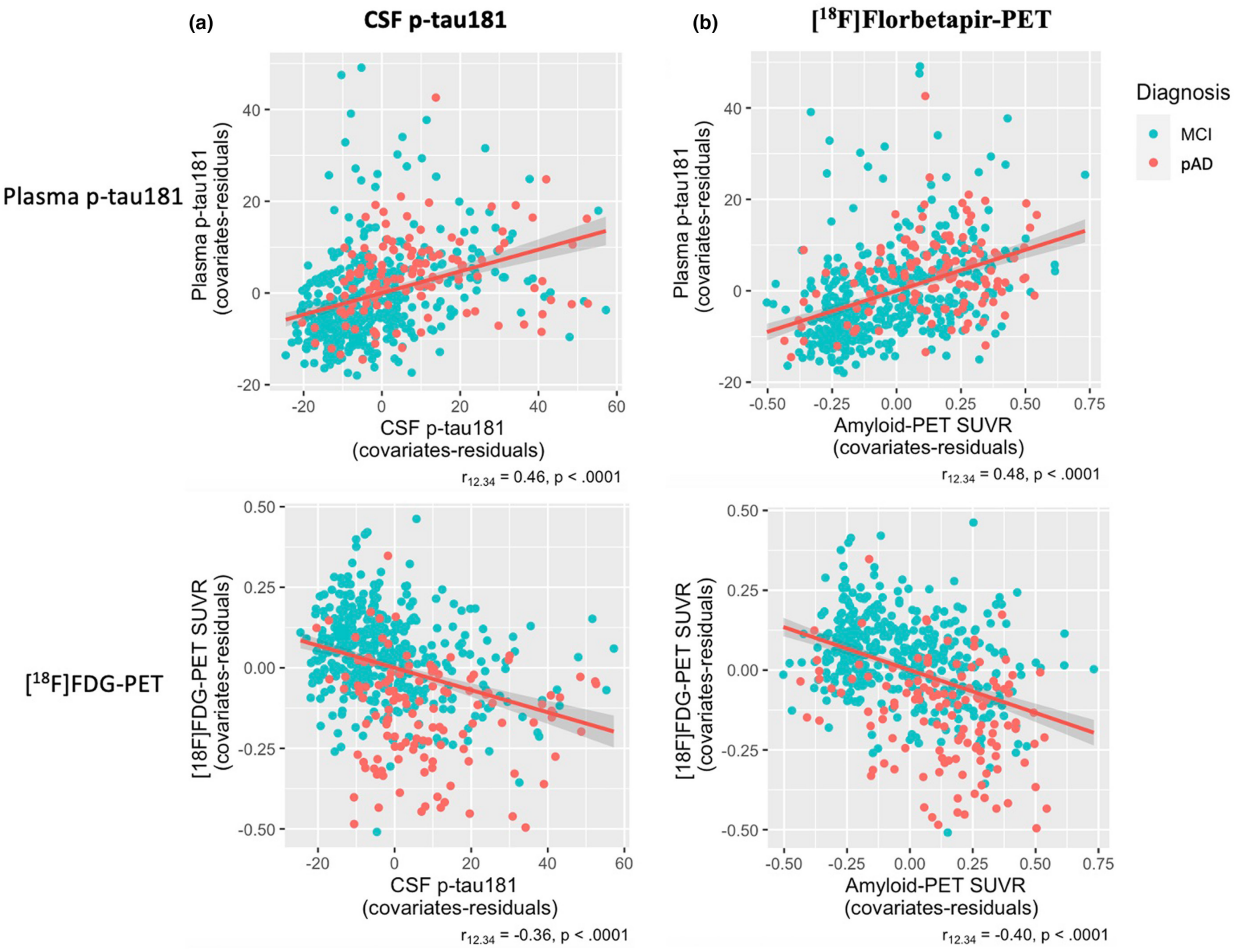
Association of plasma p‐tau181 and [^18^F]fluorodeoxyglucose (FDG) positron emission tomography (PET) standardized uptake value ratio (SUVR) with core Alzheimer disease (AD) biomarkers. Spearman partial correlation coefficient (*r*_12.34_) of (a) cerebrospinal fluid (CSF) p‐tau181 with plasma p‐tau181, CSF p‐tau181, and [^18^F]FDG SUVR and (b) Spearman partial correlation of amyloid β 42‐PET with plasma p‐tau181 and [^18^F]FDG‐PET SUVR adjusted by age and gender were computed in individuals in the cross‐sectional dataset stratified by clinical diagnosis. These measures showed significant correlation between CSF p‐tau181 and plasma p‐tau181, and between CSF p‐tau181 and [^18^F]FDG‐PET SUV. Similarly, [^18^F]florbetapir‐PET SUVR showed significant correlation with plasma p‐tau181 and with [^18^F]FDG‐PET SUVR. Association of plasma p‐tau181 and [^18^F]FDG‐PET SUVR with cognitive tests is shown. MCI, mild cognitive impairment; pAD, probable Alzheimer disease.

**FIGURE 4:**
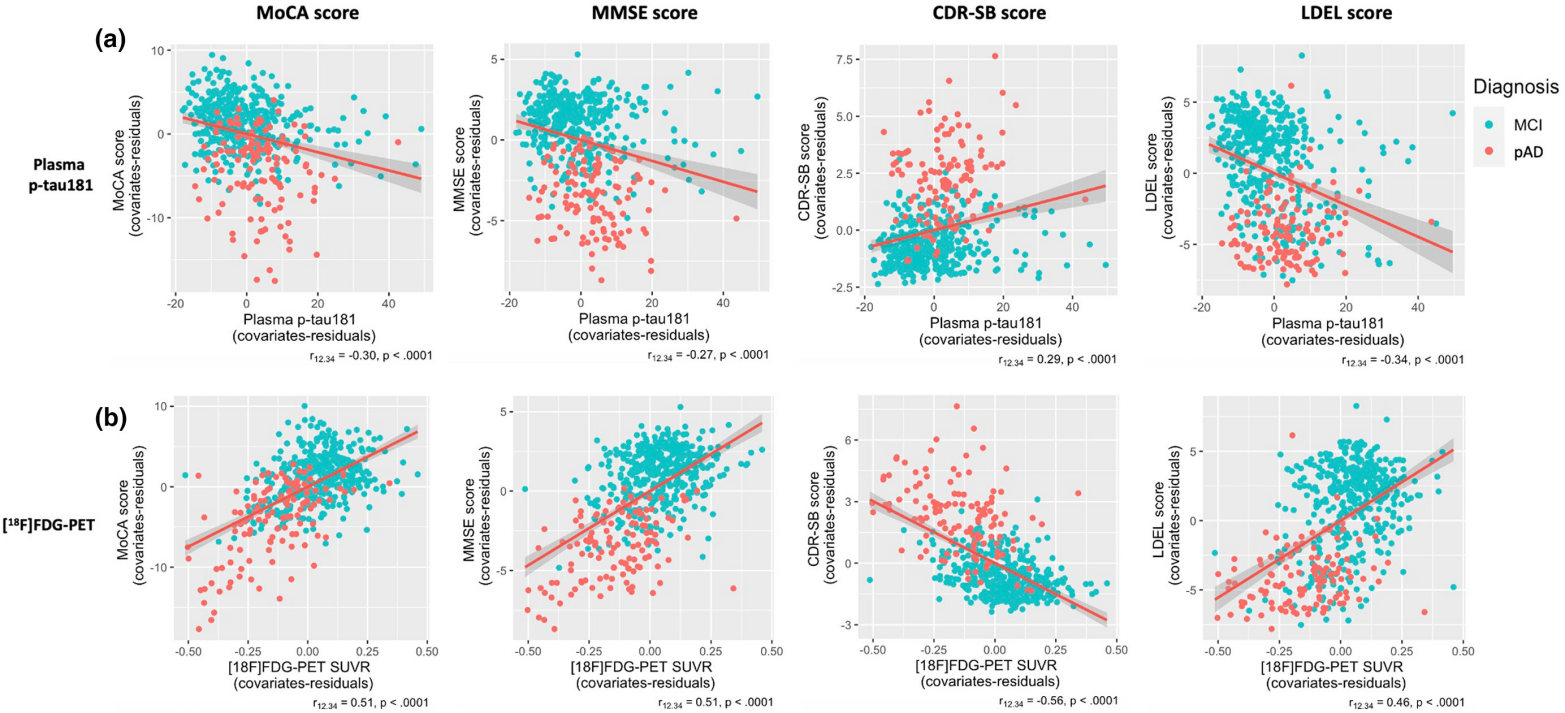
Associations of plasma p‐tau181 and [^18^F]fluorodeoxyglucose (FDG) positron emission tomography (PET) standardized uptake value ratio (SUVR) with cognitive function. Spearman partial correlation coefficient (*r*_12.34_) adjusted by sex, age, and education was determined between plasma p‐tau181 as well as [^18^F]FDG‐PET SUVR and each cognitive test. (a) Correlations of plasma p‐tau181 with Montreal Cognitive Assessment (MoCA) score, Mini‐Mental State Examination (MMSE) score, Clinical Dementia Rating Sum of Boxes (CDR‐SB) score, and logical memory delayed recall (LDEL) score. (b) Correlations of [^18^F]FDG‐PET SUVR with MoCA score, MMSE score, CDR‐SB score, and LDEL score. Both plasma p‐tau181 and [^18^F]FDG‐PET SUVR correlated with MoCA, MMSE, CDR‐SB, and LDEL scores. However, [^18^F]FDG‐PET SUVR is more closely associated with cognitive outcomes than plasma p‐tau181. MCI, mild cognitive impairment; pAD, probable Alzheimer disease.

Associations between plasma biomarker and neuroimaging biomarkers, and cognitive tests are displayed in Figures 3 and 4.

### Plasma p‐tau181 concentration and [ ^18^F]FDG‐PET SUVR are associated with core AD biomarkers

Increased concentrations of plasma p‐tau181 were partially correlated with greater CSF p‐tau181 levels (*r*_12.34_ = 0.46, *p* < 0.0001). Similarly, a significant correlation was found between [^18^F]FDG‐PET SUVR and CSF p‐tau181 levels (*r*_12.34_ = −0.36, *p* < 0.0001; Figure 3a). We also observed a negative partial correlation between [^18^F]FDG‐PET SUVR and amyloid‐PET SUVR (*r*_12.34_ = −0.48, *p* < 0.0001), whereas plasma p‐tau181 correlated positively with amyloid‐PET SUVR (*r*_12.34_ = 0.48, *p* < 0.0001; Figure 3b). The partial correlation of plasma p‐tau181 and [^18^F]FDG‐PET SUVR with CSF ratio Aβ42/t‐tau is reported in Additional File 4.

### Plasma p‐tau181 concentration and [^18^ F]FDG‐PET SUVR are associated with cognitive function

Spearman partial correlation of plasma p‐tau181 and [^18^F]FDG‐PET SUVR with neuropsychological assessments, including MMSE, MoCA, CDR‐SB, and LDEL scores adjusted by age, sex, and education, was also assessed and is shown in Figure 4. [^18^F]FDG‐PET SUVR correlated positively with MoCA score (*r*_12.34_ = 0.51, *p* < 0.0001) and with MMSE score (*r*_12.34_ = 0.51, *p* < 0.0001), whereas plasma p‐tau181 correlated negatively with lower MoCA score (*r*_12.34_ = −0.30, *p* < 0.0001) and with worse MMSE score (*r*_12.34_ = −0.27, *p* < 0.0001; Figure 4a,b). A strong negative correlation was found between [^18^F]FDG‐PET SUVR and CDR‐SB score (*r*_12.34_ = −0.56, *p* < 0.0001). However, the correlation between plasma p‐tau181 and CDR‐SB score was positive (*r*_12.34_ = 0.29, *p* < 0.0001). The association of plasma p‐tau181 and [^18^F]FDG‐PET SUVR with LDEL scores was also calculated. Whereas plasma p‐tau181 correlated negatively with LDEL score (*r*_12.34_ = −0.34, *p* < 0.0001), the correlation between [^18^F]FDG‐PET SUVR and LDEL (*r*_12.34_ = 0.46, *p* < 0.0001) score was positive (Figure 4a,b). Both plasma p‐tau181 and [^18^F]FDG‐PET SUVR correlated with poorer MoCA, MMSE, CDR‐SB, and LDEL scores; however, [^18^F]FDG‐PET SUVR was more closely associated with cognitive outcomes than plasma p‐tau181 (MoCA: *z* = 4.71, 95% CI = 0.15–0.36, *p* < 0.0001; MMSE: *z* = 5.33, 95% CI = 0.18–0.39, *p* < 0.0001; CDR‐SB: *z* = 6.17, 95% CI = 0.23–0.44, *p* < 0.0001; LDEL: *z* = 2.68, 95% CI = 0.07–0.24, *p* < 0.007).

## DISCUSSION

This study compares the diagnostic performance of plasma p‐tau181 and [^18^F]FDG‐PET for AD, as well as their association with clinical symptoms. We observed that although both plasma p‐tau181 concentrations and [^18^F]FDG‐PET SUVR were associated with AD pathophysiology measured by core AD biomarkers (CSF and Aβ‐PET), plasma p‐tau181 outperformed [^18^F]FDG‐PET in identifying individuals with AD pathophysiology. However, [^18^F]FDG‐PET was more strongly associated with neuropsychological assessments than plasma p‐tau181. Taken together, our study suggests that plasma p‐tau181 contributes to the identification of individuals with underlying AD pathophysiology.

Plasma biomarkers of AD pathophysiology are promising to transform the diagnosis of AD^16^. Although CSF and neuroimaging biomarkers are increasingly being used to support a diagnosis of AD [5, 6], these methods are limited by their invasiveness and cost, as well as by limited accessibility. Plasma biomarkers, on the other hand, offer advantages over CSF and neuroimaging assessments, as blood collection is easier to implement, more accessible, less invasive, and more cost‐effective. Our study provides evidence that plasma p‐tau181 is superior to [^18^F]FDG‐PET in identifying individuals with biomarker‐defined AD. Specifically, plasma p‐tau181 was superior to [^18^F]FDG‐PET in individuals with MCI, who may not yet have developed the pattern of hypometabolism that characterizes AD. In contrast, in individuals with pAD, plasma p‐tau181 was numerically higher but not statistically different from [^18^F]FDG‐PET for patient classification. Even though plasma p‐tau181 showed better performance in identifying biological AD biomarkers, neither plasma p‐tau181 nor [^18^F]FDG‐PET is accurate enough in isolation to detect AD in MCI and AD groups with an AUC of 0.80% or higher.

MCI is a highly heterogenous clinical syndrome, with many cases not attributable to AD [32]. The ability to identify individuals with MCI who have biological AD is critical to optimize responses to antiamyloid antibody therapy [33].

In our study, plasma p‐tau181 was better able than [^18^F]FDG‐PET to identify amyloid‐PET positivity and biological AD (defined as abnormal amyloid‐β and p‐tau181 in CSF) in individuals with MCI. These results are concordant with the superior specificity of p‐tau181 for biological AD [9, 10, 11, 12] and its close association with Aβ plaques [14].

Taken together, these results also support plasma p‐tau181 for earlier identification of biological AD than can be accomplished with [^18^F]FDG‐PET, particularly in the early clinical stages of AD, when only mild metabolic dysfunction is detectable at the group level [34]. Whereas several recent studies have performed head‐to‐head assessments of the diagnostic performance between different plasma biomarkers and amyloid‐ and tau‐PET imaging [35, 36, 37, 38], our study compared the performance of plasma p‐tau biomarker with the metabolic patterns associated with AD. Across clinical diagnoses, the capacity of plasma p‐tau181 to identify individuals with a positive CSF profile was better compared to [^18^F]FDG‐PET in the MCI group. However, there was no difference in the AD group. The performance of plasma p‐tau181 was also better than that of [^18^F]FDG‐PET to identify amyloid positivity indexed by PET in the MCI group. At the same time, there was no difference in the AD group. These results demonstrate that plasma p‐tau181 better associates with core AD pathologies than [^18^F]FDG‐PET in early symptomatic disease (MCI) when patterns of cerebral metabolic changes are not yet as pronounced as they may be in AD. [^18^F]FDG‐PET, a measure of neurodegeneration, is considered to become abnormal at late stages of AD as compared to fluid biomarkers such as p‐tau [39, 40, 41]. Therefore, in addition to the higher specificity of p‐tau181 for biological AD, it is reasonable to suggest that plasma p‐tau181 will also have higher sensitivity for AD, especially at early stages.

In agreement with recent studies [42, 43], we observed that plasma p‐tau181 and [^18^F]FDG‐PET SUVRs were associated with AD pathophysiology measured by core AD biomarkers (CSF and amyloid‐PET). We also found that higher plasma p‐tau181 were associated with a positive CSF profile and a positive amyloid‐PET result. However, we found that [^18^F]FDG‐PET was more strongly correlated to neuropsychological assessment scores than plasma p‐tau181. Although this suggests that the plasma p‐tau181 biomarker may perform less optimally as a biomarker of disease progression as compared to [^18^F]FDG‐PET, its diagnostic accuracy will be helpful for identifying individuals eligible for PET scanning to determine eligibility for disease‐modifying therapies in protocols requiring that test [34]. It should be remembered, however, that cognitive reserve can modulate the relationship between cognitive performance and [^18^F]FDG‐PET, which could on an individual basis result in discordances between imaging and clinical status [44]. The recent proliferation of plasma biomarkers, particularly p‐tau assays, has expanded the ability to identify different aspects of AD pathology [34]. In addition to identifying different aspects of AD, different plasma p‐tau assays and p‐tau isoforms have different diagnostic performances. Notably, head‐to‐head studies suggest plasma p‐tau181 is inferior to plasma p‐tau217 [35, 36, 37, 38]. Therefore, although plasma p‐tau217 is not available in the ADNI cohort at this time, we anticipate that p‐tau217 in plasma will have an even greater superiority over [^18^F]FDG‐PET for the identification of biological AD. These findings highlight the transformative nature of plasma p‐tau biomarkers for the diagnosis of AD and their future role in determining eligibility for antiamyloid therapies or for clinical trial selection.

### Limitations

Our study has limitations. An important limitation is the lack of availability of plasma p‐tau217 in the ADNI cohort. Plasma p‐tau217 has superior diagnostic performance as compared to p‐tau181 [35, 36, 37, 38], and is more closely correlated with amyloid‐β and tau pathologies [14]. Therefore, we anticipate that plasma p‐tau217 biomarkers will outperform [^18^F]FDG‐PET in the diagnosis of AD by a greater margin than reported in this study for plasma p‐tau181. A second important limitation is the highly selected nature of the ADNI cohort. Replicating the present results in a memory clinic setting (ideally with plasma p‐tau217) [37] is of great importance for determining the generalizability of the findings presented here. Related to this, our study examined hypometabolism in posterior parietal cortices, which are hypometabolic in typical AD and may be less affected in atypical clinical variants of AD. A third limitation of this study is the use of the reference standards Aβ and p‐tau181 in CSF, which have high imperfect agreement with PET and with neuropathology. Although these reference standards are used in the clinical diagnosis of AD, replication of these findings using either amyloid‐PET and tau‐PET or neuropathology will help increase confidence in these findings. Another important limitation of our study is that chronic kidney disease may affect concentrations of plasma biomarkers [13], and ratio measurements to correct this potential bias [45] are not yet available in the ADNI cohort.

## CONCLUSIONS

In conclusion, our study provides evidence that plasma p‐tau181 measurements outperform [^18^F]FDG‐PET in the identification of biological AD in individuals with MCI. [^18^F]FDG‐PET, however, had stronger correlation with clinical symptoms and may be a better biomarker of disease progression.

## CONFLICT OF INTEREST STATEMENT

P.R.‐N. is a member of the CIHR‐CCNA Canadian Consortium of Neurodegeneration in Aging and Colin J. Adair Charitable Foundation. S.G. serves as a scientific advisor for Cerveau and Enigma US. E.R.Z. serves on the scientific advisory board of Next Innovative Therapeutics. H.Z. has served on scientific advisory boards and/or as a consultant for Abbvie, Acumen, Alector, Alzinova, ALZPath, Annexon, Apellis, Artery Therapeutics, AZTherapies, CogRx, Denali, Eisai, Nervgen, Novo Nordisk, Optoceutics, Passage Bio, Pinteon Therapeutics, Prothena, Red Abbey Labs, reMYND, Roche, Samumed, Siemens Healthineers, Triplet Therapeutics, and Wave, has given lectures at symposia sponsored by Cellectricon, Fujirebio, Alzecure, Biogen, and Roche, and is a cofounder of Brain Biomarker Solutions in Gothenburg, which is a part of the GU Ventures Incubator Program. The other authors declare no conflict of interest.

## FUNDING INFORMATION

This research is supported by the Weston Brain Institute, Canadian Institutes of Health Research (CIHR; MOP‐11‐51‐31; RFN 152985, 159815, 162303), Canadian Consortium of Neurodegeneration and Aging (CCNA; MOP‐11‐51‐31 ‐team 1), Alzheimer's Association (NIRG‐12‐92,090, NIRP‐12‐259245), Brain Canada Foundation (CFI Project 34874; 33397), and Fonds de Recherche du Québec–Santé (Chercheur Boursier, 2020‐VICO‐279314). T.A.P., P.R.‐N., and S.G. are members of the CIHR‐CCNA Canadian Consortium of Neurodegeneration in Aging and Colin J. Adair Charitable Foundation. Data collection and sharing for this project were funded by ADNI (NIH grant U01 AG024904) and DOD ADNI (Department of Defense award number W81XWH‐12‐2‐0012). ADNI is funded by the National Institute on Aging and the National Institute of Biomedical Imaging and Bioengineering, and through contributions from the following: AbbVie, Alzheimer's Association, Alzheimer's Drug Discovery Foundation, Araclon Biotech, BioClinica, Biogen, Bristol‐Myers Squibb, CereSpir, Eisai, Elan Pharmaceuticals, Eli Lilly and Company, EuroImmun, F. Hoffmann‐La Roche and its affiliated company Genentech, Fujirebio, GE Healthcare, IXICO, Janssen Alzheimer Immunotherapy Research & Development, Johnson & Johnson Pharmaceutical Research & Development, Lumosity, Lundbeck, Merck & Co., Meso Scale Diagnostics, NeuroRx Research, Neurotrack Technologies, Novartis Pharmaceuticals Corporation, Pfizer, Piramal Imaging, Servier, Takeda Pharmaceutical Company, and Transition Therapeutics. The CIHR also provides funds to support ADNI clinical sites in Canada. Private sector contributions are facilitated by the Foundation for the NIH (fnih.org). The grantee organization is the Northern California Institute for Research and Education, and the study is coordinated by the AD Cooperative Study at the University of California, San Diego. ADNI data were disseminated by the Laboratory for NeuroImaging at the University of Southern California. H.Z. is a Wallenberg Scholar supported by grants from the Swedish Research Council (#2022–01018 and #2019–02397), the European Union's Horizon Europe research and innovation programme under grant agreement 101053962, Swedish State Support for Clinical Research (#ALFGBG‐71320), the Alzheimer Drug Discovery Foundation, USA (#201809–2016862), the AD Strategic Fund and the Alzheimer's Association (#ADSF‐21‐831,376‐C, #ADSF‐21‐831,381‐C, and #ADSF‐21‐831,377‐C), the Bluefield Project, the Olav Thon Foundation, the Erling‐Persson Family Foundation, Stiftelsen för Gamla Tjänarinnor, Hjärnfonden, Sweden (#FO2022‐0270), the European Union's Horizon 2020 research and innovation programme under Marie Skłodowska‐Curie grant agreement 860197 (MIRIADE), the European Union Joint Programme–Neurodegenerative Disease Research (JPND2021‐00694), the National Institute for Health and Care Research University College London Hospitals Biomedical Research Centre, and the UK Dementia Research Institute at UCL (UKDRI‐1003).

## Supporting information


**Data S1**.

## Data Availability

The data used in this article can be found in the ADNI database (https://adni.loni.usc.edu/).
