# Supplementary material for: Plasma phosphorylated tau181 outperforms [ 18F] fluorodeoxyglucose positron emission tomography in the identification of early Alzheimer disease
Source: Eur J Neurol. 2024 Oct 24;31(12):e16255. doi: 10.1111/ene.16255 (PMC11555153; doi:10.1111/ene.16255)
Supplement: Supplementary file 1 — Data S1. [file ENE-31-e16255-s001.docx]

**Additional files:**

**
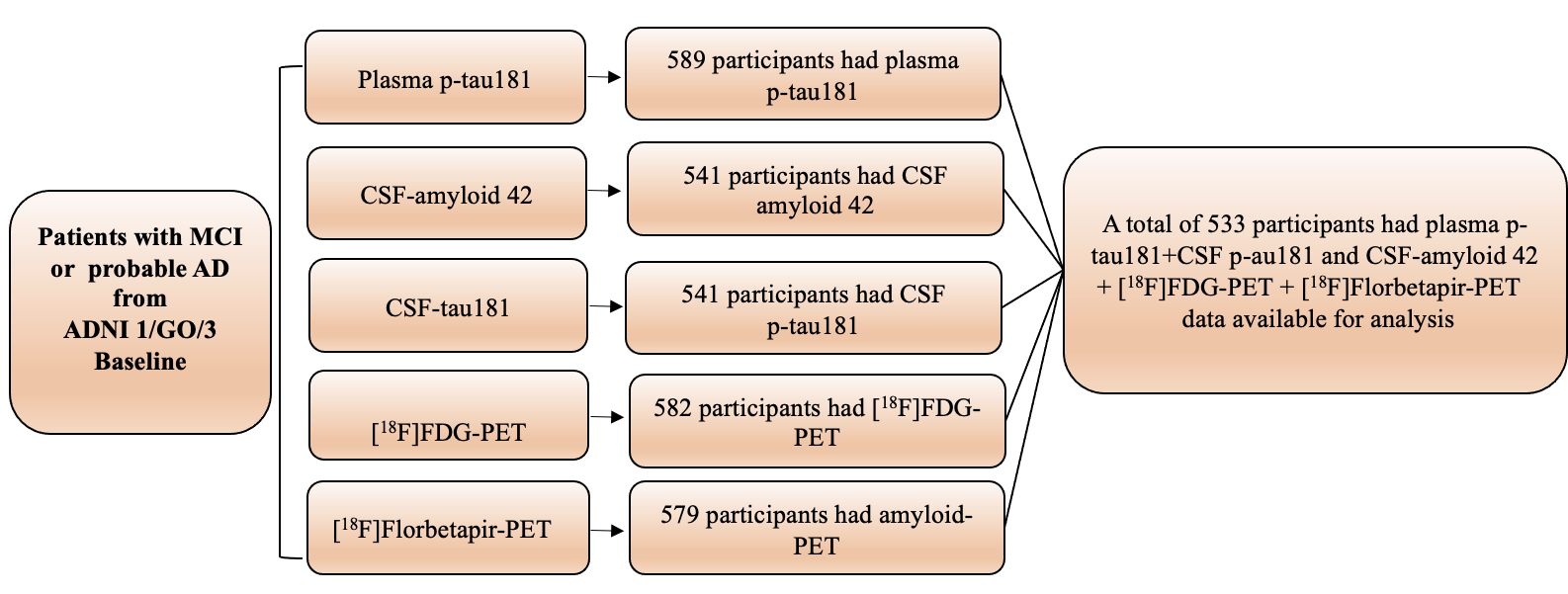
**

***Additional file 1:*** *Distribution of plasma p-tau181 according to clinical group and amyloid-PET status.*


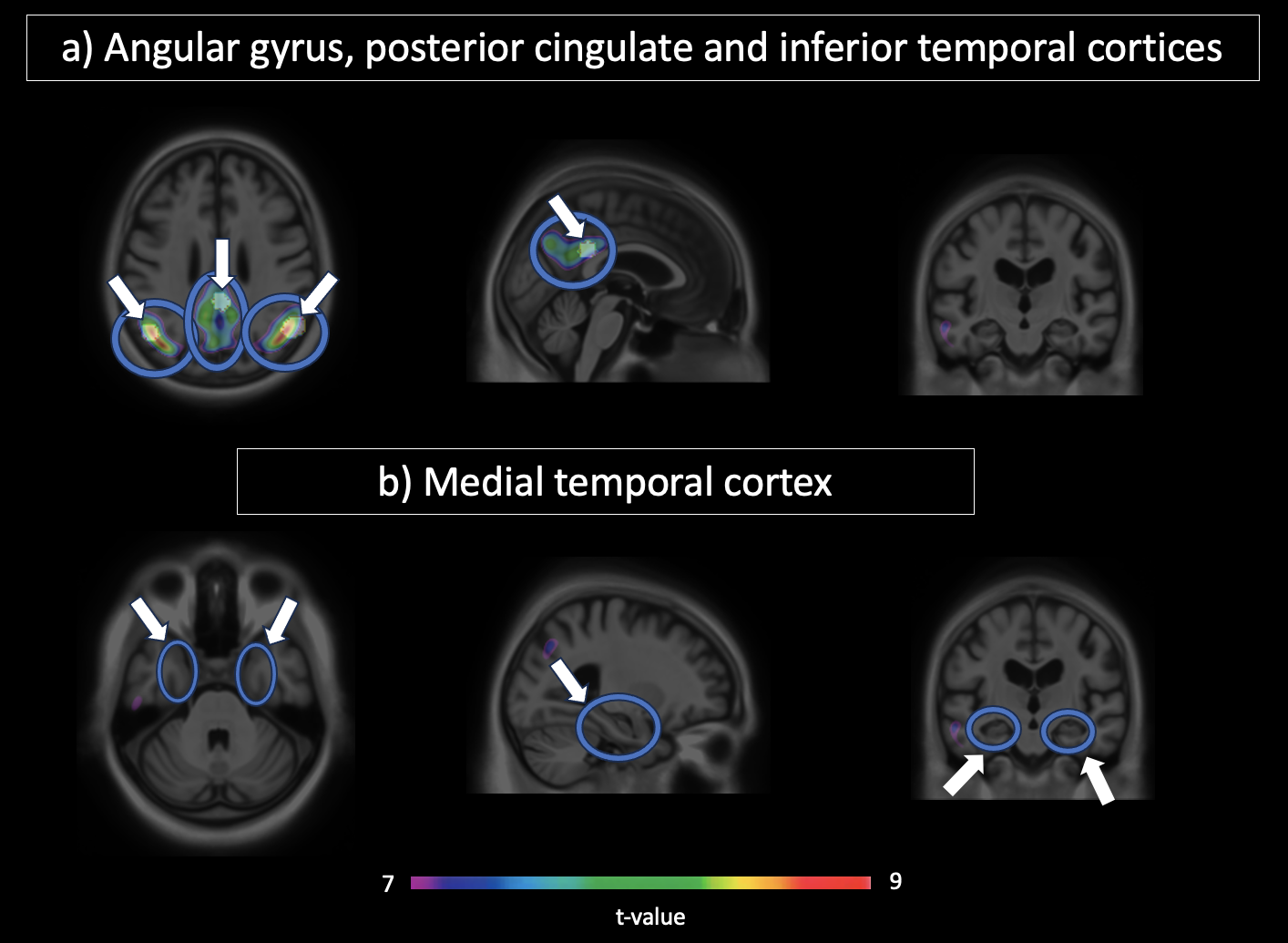


***Additional file 2.*** *Voxel-based regression analyses of [^18^F]FDG-PET uptake retention between amyloid positive and negative groups. Voxel-based analyses were corrected for age, gender and education, and were corrected for multiple comparisons using random field theory at P < 0.001.*

***Additional file 3:*** *Distribution of plasma p-tau181 according to clinical group and amyloid-PET status.*


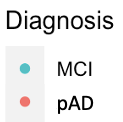

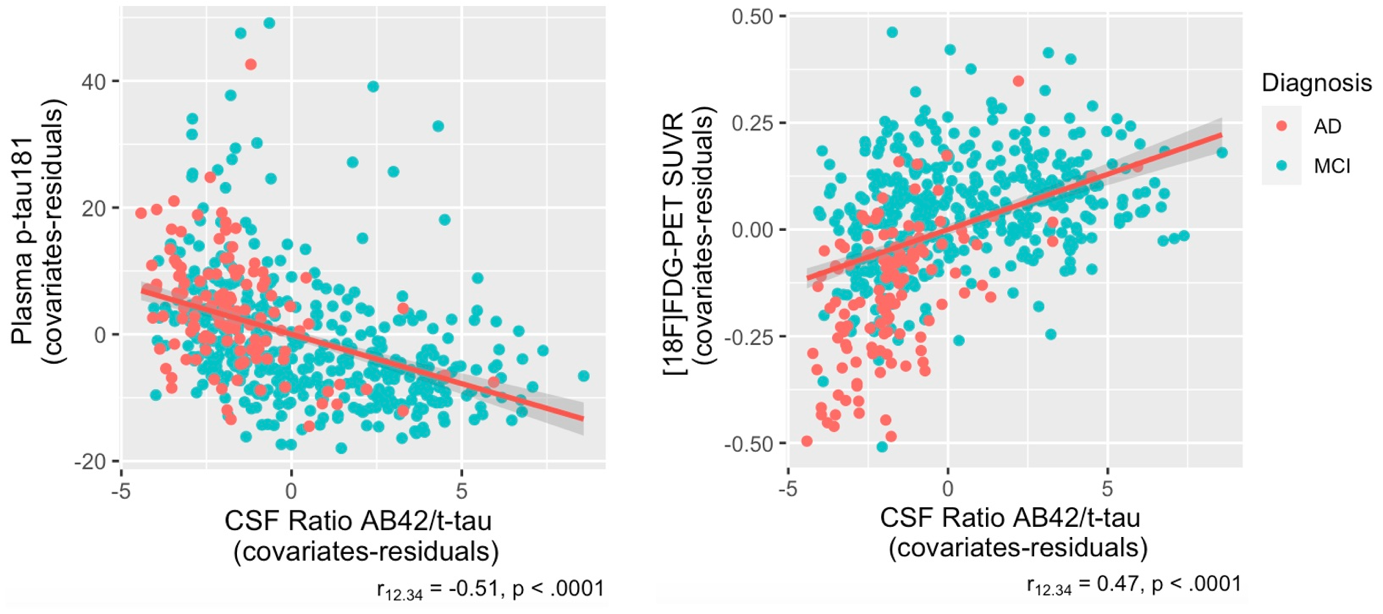


***Additional file 4:*** *Association of plasma p-tau 181, and [^18^F]FDG-PET SUVR with CSF Aβ42/t-tau ratio adjusted by age and sex.*
